# Tumor cells-derived conditioned medium induced pro-tumoral phenotypes in macrophages through calcium-nuclear factor κB interaction

**DOI:** 10.1186/s12885-022-10431-8

**Published:** 2022-12-19

**Authors:** Yuexin Zhang, Ziqi Zhang, Lei Chen, Xiuyue Zhang

**Affiliations:** 1 Key Laboratory of Bio-Resources and Eco-Environment, Ministry of Education, College of Life Science, Sichuan University, Chengdu, 610064 China; 2 Gastric and Colorectal Surgery Division, Department of General Surgery, Daping Hospital, Army Medical University, No. 10, Changjiangzhilu, Daping, Yuzhong District Chongqing, 400042 China; 3 Laboratory of Aging Research and Cancer Drug Target, State Key Laboratory of Biotherapy, National Clinical Research Center for Geriatrics, West China Hospital, Sichuan University, No. 17, Block 3, Southern Renmin Road, Chengdu, Sichuan 610041 People’s Republic of China

**Keywords:** LL-2, CMT-64 macrophages, NF-κB, Calcium, RNA-seq

## Abstract

**Background:**

The malignant behaviors of lung cancers are affected by not only cancer cells but also many kinds of stromal cells in tumor microenvironment (TME), including macrophages. Macrophages have been proven to extensively influence tumor progression through several mechanisms, among which switching of macrophages from pro-inflammatory phenotypes (M1-like) to anti-inflammatory phenotypes (M2-like) mediated by transcription factors such as nuclear factor κB (NF-κB) is the most crucial event. The regulation of NF-κB has been well studied, however some details remain fuzzy.

**Methods:**

Mouse primary bone marrow-derived macrophages (BMDMs) were cultured in Lewis lung carcinoma cell line LL-2-derived conditioned medium (LL-2-CM). Proliferation, migration, and polarization of BMDMs were tested by CCK8, scratch test, transwell, and flow cytometry. Secretion of several cytokines were detected by ELISA or cytometric bead array. To further explore the underlying mechanisms, BMDMs cultured in LL-2-CM were harvested for RNA-seq. Cytosolic calcium was detected by calcium probe Fluo-4-AM. Western blot was applied to exam the activation of NF-κB signal. BAPTA-AM was applied to sequestrate cytosolic calcium to further investigate the relationship between calcium and NF-κB signal. The polarization, calcium alteration, and NF-κB signal activation were further validated in BMDMs treated by CMT-64-derived conditioned medium (CMT-64-CM).

**Results:**

LL-2-CM promoted proliferation, migration, and M2-like polarization of BMDMs and inhibited M1-like polarization of BMDMs. However two pro-inflammatory cytokines, interleukin-6 (IL-6) and tumor necrosis factor-$\mathrm{\alpha }$ (TNF-$\mathrm{\alpha }$) were secreted. RNA-seq indicated that LL-2-CM activated both canonical and non-canonical NF-κB signal in BMDMs. Western blot showed that canonical NF-κB was temporarily elicited and attenuated at 24 h, while non-canonical NF-κB was consistently activated. At the same time, expression of genes that regulate cytosolic calcium ion concentration were down regulated, which caused diminution of cytosolic calcium in BMDMs treated with LL-2-CM. The decreased cytosolic calcium, M2-like polarization, and NF-κB activation was also observed in CMT-64-CM treated BMDMs. On the contrary, elevated cytosolic calcium was observed during M1-like polarization of BMDMs elicited by lipopolysaccharide (LPS). Interestingly, administration of calcium chelator, BAPTA-AM, impeded activation of canonical NF-κB and expression of M1-like marker induced by LPS, which further confirmed the relationship between cytosolic calcium and canonical NF-κB signal.

**Conclusions:**

In summary, lung cancer cell-derived conditioned medium promoted migration, proliferation, and M2-like polarization of BMDMs. The suppressed M1-like polarization was achieved through mitigating canonical NF-κB pathway via diminishing cytosolic calcium concentration. As far as we know, our work firstly revealed that cytosolic calcium is the key during inhibition of canonical NF-κB and M1-like polarization in macrophages by tumor cells.

**Supplementary Information:**

The online version contains supplementary material available at 10.1186/s12885-022-10431-8.

## Background

Macrophages are a group of multi-facetted cells that exist in almost all tissues. The alterations of macrophage functions was underpinned by diverse transcriptomes in response to different stimuli [1]. Briefly, macrophages are classified into two main groups. M1-like macrophages are pro-inflammatory cells stimulated by ligands of Toll-like receptors (TLRs), interferon gamma (IFN-γ), and components of micro-organisms [2–5]. NF-κB, the down-stream transcriptome factor of TLR signals, has been reported to mediate M1-like polarization of macrophages in TME [6]. Selective degradation of NF-κB p65, a component of canonical NF-κB signal, reversed M1-like phenotypes of macrophages into M2-like phenotypes [6]. The markers of M1-like macrophages include major histocompatibility complex class II (MHC-II), inducible nitric oxide (iNOS), and CD86. On the contrary, anti-inflammatory M2-like macrophages, characterized to be CD206^+^ and arginase-1^+^, are induced by interleukin-4 (IL-4), interleukin-10 (IL-10), and transforming growth factor beta (TGF-β) [2, 7–10]. In TME, macrophages always show M2-like phenotypes, although they may express M1-like phenotypes at early stage [11]. The M2-like polarization of macrophages is mediated by cross-talk between tumor cells and macrophages [12, 13]. M2-like macrophages inhibit anti-tumor immunity of T cells and nature killer cells (NKs), thus reasonable strategies of cancer therapies are reprogramming M2-like macrophages into M1-like macrophages by TLR ligands (such as classical TLR4 stimulator LPS) [14] and mitigating macrophage recruitment and polarization in TME by colony stimulating factor receptor (CSFR) signal inhibitors [15]. Although many research works have illustrated cross-talks between tumor cells and macrophages, the high-throughput analysis of the signal changes and the related mechanisms in macrophages treated by LL-2-CM remains limited.

In this research, LL-2-CM promoted proliferation, migration, and M2-like polarization of BMDMs, while pro-inflammatory IL-6 and TNF-$\mathrm{\alpha }$ was also secreted. RNA-seq demonstrated that both canonical and non-canonical NF-κB signals were activated and many GO terms mediating cytosolic calcium ion homeostasis were down regulated after LL-2-CM treatment. Using cytosolic calcium probe, Fluo-4-AM, we found that LL-2-CM treatment decreased cytosolic calcium concentration. Since cytosolic calcium has been reported to stimulate NF-κB signal in lymphocytes [16], we tested the activation of NF-κB signals in LL-2-CM treated BMDMs and the relationship between calcium and canonical NF-κB signal in BMDMs using western blot. Our data revealed that canonical NF-κB signal was temporarily activated within 24 h, while non-canonical NF-κB signal was consistently activated. The decreased cytosolic calcium concentration was possibly responsible for the mitigated NF-κB signal, because chelating cytosolic calcium via BAPTA-AM obviously suppressed NF-κB activation and the down-stream M1-like polarization of macrophages induced by LPS. Additionally, similar M2-like polarization and cross-talk between cytosolic calcium and NF-κB signal were observed in CMT-64-CM treated BMDMs. Our work provided an overall view of the effects of lung cancer cell conditioned medium on BMDMs and highlighted cytosolic calcium attenuation to be the potential cause of the diminished NF-κB signal and the promoted M2-like polarization in macrophages.

## Methods

### Extraction and culture of primary BMDMs

Wild type 6–8 weeks C57BL/6 mice were purchased from Beijing Vital River Laboratory Animal Technology and fed in specific pathogen free condition with suitable light–dark cycle and adequate water and forage. Our study was reviewed and approved by the Institutional Animal Care and Use Committee of Sichuan University (Chengdu, Sichuan, China). All the experiments were conducted in compliance with the protocols approved by the Institutional Animal Care and Use Committee of Sichuan University (Chengdu, Sichuan, China). Our study was conducted in accordance with ARRIVE guidelines.

For each experiment, mice were anaesthetized using isoflurane and sacrificed. Three or four mice were euthanasia to isolate bilateral tibias and femurs in sterile condition. Bone marrow was flushed out by RPMI-1640 medium (C11875500BT, Gibco). The bone marrow was centrifuged and resuspended with red blood cell lysis buffer. Then the cell pallets were centrifuged and resuspended with RPMI-1640 medium to remove red blood cell lysis buffer. Next, the cells were filtrated by 70 $\mathrm{\mu m}$ cell strainers (352,350, Falcon) and counted. Finally, the bone marrow cells were centrifuged and resuspended by RPMI-1640 medium supplied with 10 $\mathrm{\%}$ FBS (10,091,148, Gibco), 1 $\mathrm{\%}$ penicillin/streptomycin (SV30010, HyClone), and 20 ng/mL M-CSF (CB34, Novoprotein). For 6-well (3506, Corning)/12-well (3513, Corning)/96well plates (310,109,008, LabServ), $2\times {10}^{6}$/$1\times {10}^{6}$/$1\times {10}^{5}$ cells were added per wells respectively. Two or three days later, the suspended cells were removed and the adherent cells were used for the appropriate experiment.

### Cell culture and conditioned medium collection of lung cancer cell lines

LL-2 and CMT-64 cell line were obtained from American Tissue Type Culture Collection (ATCC). LL-2 or CMT-64 cells were cultured in DMEM (C11995500BT, Gibco) supplied with 10 $\mathrm{\%}$ FBS, 1 $\mathrm{\%}$ penicillin/streptomycin. For conditioned medium collection, when LL-2 or CMT-64 cell density reached about 80 $\mathrm{\%}$, the medium was replaced with new DMEM supplied with 10 $\mathrm{\%}$ FBS, 1 $\mathrm{\%}$ penicillin/streptomycin. After 24 h, the conditioned medium was harvested and filtrated by 0.22 $\mathrm{\mu m}$ filter (SLCPR33RB, Millipore) and stored at -20 $\mathrm{^\circ{\rm C} }$.

### CCK8 assay

BMDMs were seeded into 96-well plates. Two to three days later, RPMI-1640 medium was removed and medium containing 0 $\mathrm{\%}$, 25 $\mathrm{\%}$, 50 $\mathrm{\%}$, and 100 $\mathrm{\%}$ volume of LL-2-CM was used to culture BMDMs for further 48 h. The remaining volume of the medium was completed by RPMI-1640 medium that used for culturing BMDMs. Then the BMDMs were treated with CCK8 (HY-K0301, MedChemExpress) referring to manufacture’s instructions. Finally, microplate reader at 450 nm was used to read the plates.

### BCA

Protein concentrations in mediums were measured by BCA Protein Assay Kit (23,225, Thermo Fisher) according to manufacturer’s instructions. Briefly, 5 $\mathrm{\mu l}$ diluted medium were added into 100 $\mathrm{\mu l}$ mixture of reagent A and reagent B in a 96-well plate. At the same time, a sequence of dilution of standard bovine serum albumin solution were used to create a standard curve. The 96-well plate were incubated in 37 $\mathrm{^\circ{\rm C} }$ for 30 min before read by microplate reader at 562 nm.

### Scratch test

BMDM monolayer in 6-well plate was scratched with 10 ml pipette. The cells were washed once to remove suspension cells before photographed. Equal volume of LL-2-CM or new DMEM medium was added into RPMI-1640 medium that used for culturing BMDMs. After 24 h, the scratches were photographed again at the same place to evaluate cell migration. The width of scratches were measured by Image J. The scratch closure rates were calculated by $100\mathrm{\%}\times \left({width}_{0h}-{width}_{24h}\right)/{width}_{0h}$.

### Transwell test

BMDMs were seeded into transwell (3422, Corning) with 8 $\mathrm{\mu m}$ pores. For each transwell, $5\times {10}^{5}$ BMDMs were loaded into the up chamber and DMEM medium containing 50 $\mathrm{\%}$ LL-2-CM were loaded into the down chamber. The transwells were stained and photographed after 12 h. The cells in different visual fields were counted.

### Flow cytometry

BMDMs were cultured in 12-well plates. Two to three days later, equal volume of LL-2-CM, CMT-64-CM, or new DMEM medium was added into RPMI-1640 medium that used for culturing BMDMs for further 12 h, 24 h, or 48 h. For LPS stimulation, 20 ng/ml LPS (L2880, Sigma-Aldrich) was added into RPMI-1640 medium with or without 10 $\mathrm{\mu M}$ BAPTA-AM (HY-100545, MedChemExpress) to culture BMDMs for further 24 h. Cells were collected and stained by antibodies in 4 $\mathrm{^\circ{\rm C} }$ for 30 min. Anti-CD45 BV421 (103,133, Biolegend), anti-CD206 APC (141,707, Biolegend), anti-CD206 PE (141,706, Biolegend), anti-MHC-II BV510 (107,635, Biolegend), and anti-MHC-II BV711 (107,643, Biolegend) were used. At the same time, LIVE/DEAD™ Fixable Near-IR Dead Cell Stain Kit (L34975, Thermo Fisher) was added to exclude non-specific staining. After staining, the cells were washed once by PBS and analyzed by ACEA NovoCyte (ACEA Biosciences). The gating strategies for our experiments have been shown in supplementary Fig. 1.

### ELISA

Concentrations of IL-4 in medium were measured by ELISA kit (88–7044-22, Thermo Fisher) according to manufacturer’s instructions. Briefly, 96-well plates were coated by capture antibody overnight. After blocking, the wells were loaded with our medium or a sequence of dilution of standard IL-4 solution overnight. Then the wells were incubated by detection antibody followed by HRP. Finally TMB solution followed by Stop solution were used to detect the remaining HRP in each wells. The plates were read by microplate reader at 450 nm and 562 nm. The concentrations of IL-4 in medium were calculated referring to the standard curve.

### Cytometric bead array

Concentrations of IL-1 $\upbeta$, IL-6, IL-10, and TNF-$\mathrm{\alpha }$ in medium were measured by cytometric bead array (560,232, 558,301, 558,300, and 558,299, BD Biosciences) according to manufacturer’s instructions. Briefly, medium was incubated with capture beads and detection reagent successively. The APC, APC-Cy7, and PE information of the beads were collected by ACEA NovoCyte (ACEA Biosciences). The concentration of each cytokine was calculated referring to the standard curve.

### RNA-seq

BMDMs were cultured in 6-well plates. Two days later, equal volume of LL-2-CM or new DMEM medium was added into RPMI-1640 medium that used for culturing BMDMs. After 24 h, BMDMs were harvested and lysed in TRIzol (Invitrogen). Novogene Bioinformatics Institute, Beijing, China, performed the extraction and sequencing of RNA. The raw reads were processed with adapter trimming and reads filtering by trim galore version 0.5.0 to ensure data quality. FastQC version 0.11.5 was used to generate quality reports. The reads were mapped to Mus_musculus.GRCm38.dna.toplevel.fa and annotated with Mus_musculus.GRCm38.98.gtf.

To identify differentially expressed genes (DEGs) between two groups, fold change (FC) was calculated. The cut-off number of log2FC was set to be 1. The DEGs were further annotated using Gene Ontology (GO) and Kyoto Encyclopedia of Genes and Genomes (KEGG) database.

### Cytosolic calcium evaluation

For real-time cytosolic calcium measurement, BMDMs in 96-well plates were incubated in RPMI-1640 medium containing 2.5 $\mathrm{\mu M}$ Fluo-4-AM (F14217, Invitrogen) for 30 min. Then the RPMI-1640 medium containing Fluo-4-AM was replaced with RPMI-1640 medium with/without 50 $\mathrm{\%}$ volume of LL-2-CM. The plate was read using fluorescent microplate reader in 37 $\mathrm{^\circ{\rm C} }$ for 350 min with the excitation wavelength at 488 nm and the emission wavelength at 530 nm. The results were shown as Fluo-4 $\Delta F/{F}_{0}$ indicating the relative changes referring to the initial fluorescence intensity.

For cytosolic calcium measurement by flow cytometry, BMDMs in 12-well plates were incubated in RPMI-1640 medium with/without 50 $\mathrm{\%}$ volume of LL-2-CM or CMT-64-CM for 48 h. Then the BMDMs were stained by 2.5 $\mathrm{\mu M}$ Fluo-4-AM for 30 min. After staining, the cells were harvested and washed once by PBS and analyzed by ACEA NovoCyte.

### Western blot assay

BMDMs in 12-well plates were incubated in RPMI-1640 medium with/without 50 $\mathrm{\%}$ volume of LL-2-CM or CMT-64-CM for desired time. For cytosolic calcium chelating, BMDMs were previously incubated in 10 $\mathrm{\mu M}$ BAPTA-AM for 30 min, then the cells were stimulated by 500 ng/ml LPS, 50 $\mathrm{\%}$ volume of LL-2-CM, or 50 $\mathrm{\%}$ volume of CMT-64-CM for 2 h. After incubation, the cells were lysed by RIPA buffer (P0013B, Beyotime). The cell lysates were separated in 7.5 $\mathrm{\%}$ or 10 $\mathrm{\%}$ gels and transferred to PVDF membrane (GVWP04700, Millipore). The membranes were probed by desired antibodies. Antibodies used herein include rabbit anti-p-p65 (3033S, Cell Signaling), rabbit anti-p65 (8242S, Cell Signaling), rabbit anti-p-RelB (5025S, Cell Signaling), rabbit anti-RelB (4922S, Cell Signaling), rabbit anti-p-IKK (2697S, Cell Signaling), rabbit anti-IKK $\mathrm{\alpha }$ (2682S, Cell Signaling), rabbit anti-IKK $\upbeta$ (8943S, Cell Signaling), rabbit anti-p100/p52 (4882S, Cell Signaling), and mouse anti-$\upbeta$-actin (sc-47778, Santa Cruz). The gray scales were calculated by Image J. Gray scales of each proteins were standardized by respective $\upbeta$-actin. The standardized gray scales of each proteins in different time points or treatment groups were compared with the initial time point or blank group.

### Statistics

GraphPad Prism 8 was used to draw the statistical graphics. Student t-test was used to compare differences between two groups. One-way ANOVA followed by tukey or sidak test was used to compare differences among three or more groups. *P* < 0.05 was considered to be significant.

## Results

### LL-2-CM promoted cell proliferation and migration of BMDMs

**Fig. 1:**
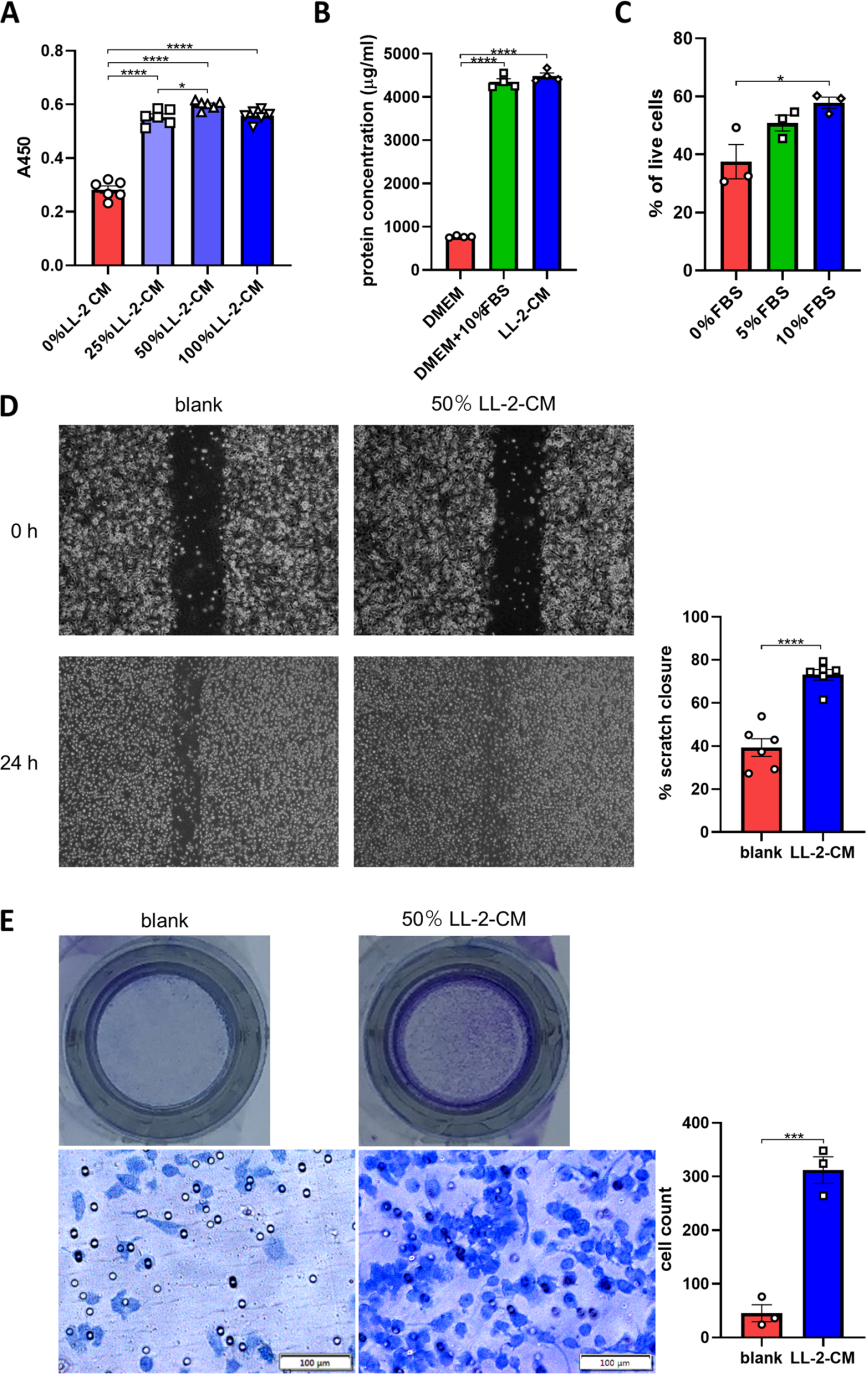
LL-2-CM promoted cell proliferation and migration of BMDMs. BMDMs were cultured in different concentrations of LL-2-CM. Viable cells were detected by CCK8 **A**. Protein concentrations in different culture medium **B** and viability of BMDMs in different concentration of FBS **C** was tested to verify the optimal LL-2-CM treatment method. *P* values were calculated by one-way ANOVA and Tukey test. Monolayer of BMDMs were scratched and cultured in 50 $\mathrm{\%}$ LL-2-CM. Pictures were taken at the same field of vision before and after 24 h. The scratch closure rates were calculated **D**. BMDMs were loaded in the up chamber of transwells and 50 $\mathrm{\%}$ LL-2-CM was loaded in the down chamber of transwells for 12 h. The cells crossing through the transwell membrane were stained and counted **E**. *P* values were calculated by Student’s t-test

It has been known that macrophages in tissues are maintained by both local proliferation and recruitment from blood monocyte [1]. In order to explore whether LL-2-CM was able to enhance proliferation of BMDMs, we used different concentrations of LL-2-CM to culture BMDMs for 48 h. Then CCK8 was applied to compare the amount of viable cells in each group. LL-2-CM significantly augmented cell proliferation in dose-dependent manner (Fig. 1A). Interestingly, this effect reached maximum in 50 $\mathrm{\%}$ volume of LL-2-CM, which was even slightly higher than 100 $\mathrm{\%}$ LL-2-CM implying this ratio of LL-2-CM and fresh RPMI-1640 was optimal for BMDM proliferation. The total protein concentrations in DMEM, DMEM supplied with FBS, and LL-2-CM were measured. The majority of protein in culture medium was provided by FBS, which was not significantly consumed by LL-2 (Fig. 1B). Additionally, reducing FBS in culture medium mitigated cell survival of BMDMs determined by flow cytometry (Fig. 1C). Thus we chose fresh medium supplied with 10 $\mathrm{\%}$ FBS and 50 $\mathrm{\%}$ cancer cell conditioned medium in the following experiments.

Migration is one of the indicators to evaluate the recruitment ability of cells in vitro [17]. Scratch test and transwell test were applied to figure out whether LL-2-CM promoted cell migration of BMDMs. LL-2-CM significantly enhanced cell migration of BMDMs (Fig. 1D, E). Together, our data showed that LL-2-CM was able to facilitate cell proliferation and migration of BMDMs.

### LL-2-CM inhibited M1-like polarization and promoted M2-like polarization of BMDMs

**Fig. 2:**
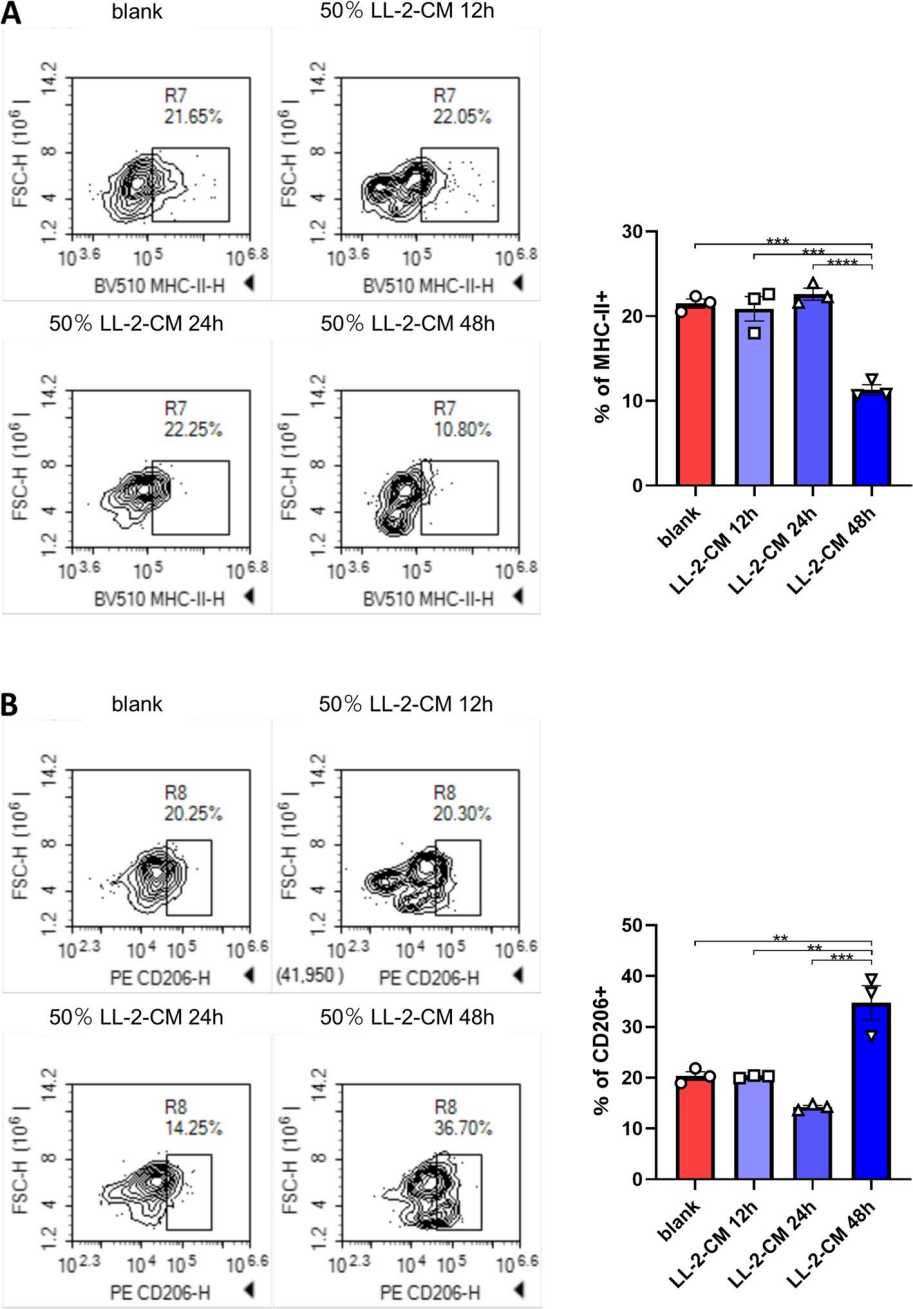
LL-2-CM inhibited M1-like polarization and promoted M2-like polarization of BMDMs. BMDMs cultured with or without LL-2-CM were stained by anti-MHC-II **A** and anti-CD206 **B**. *P* values were calculated by one-way ANOVA and Tukey test

To further investigate the influence of LL-2-CM on the phenotypes of BMDMs, we used flow cytometry to test the cell polarization of BMDMs treated by LL-2-CM. The marker of M1-like polarization, MHC-II, was significantly down regulated by LL-2-CM (Fig. 2A), while the marker of M2-like polarization, CD206, was significantly up-regulated (Fig. 2B). Interestingly, MHC-II was slightly elevated and CD206 was slightly mitigated in 24 h group (Fig. 2A, B) although the alterations were not significant. Our results demonstrated that LL-2-CM was able to induce anti-inflammatory phenotype of BMDMs.

### LL-2-CM promoted production of pro-inflammatory cytokines from BMDMs

**Fig. 3:**
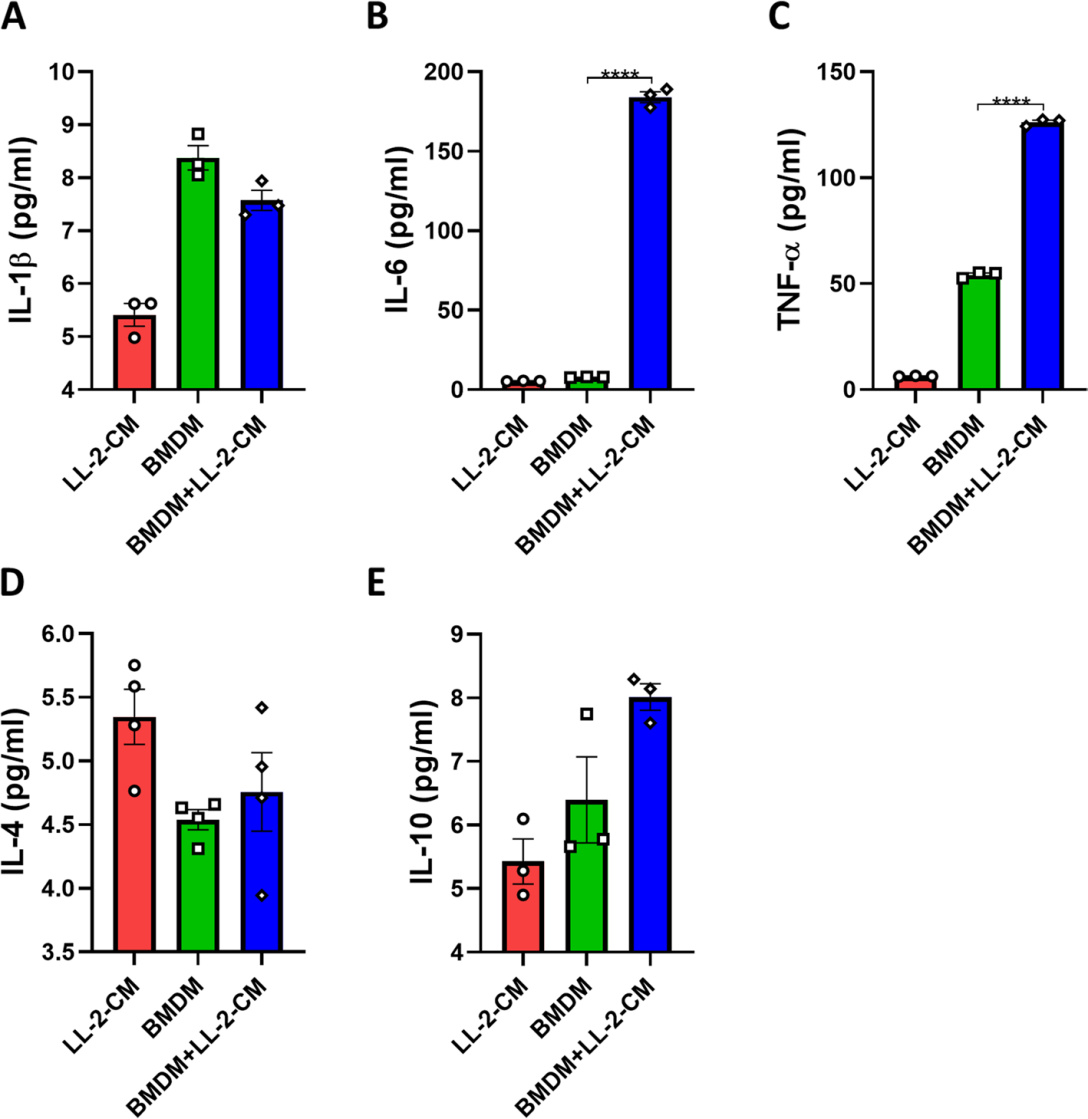
LL-2-CM promoted secretion of IL-6 and TNF-$\mathrm{\alpha }$. Cytometric bead array was applied to measure the concentrations of IL-1 $\upbeta$ **A**, IL-6 **B**, TNF-$\mathrm{\alpha }$ **C**, and IL-10 **E** and ELISA was applied to measure the concentrations of IL-4 **D** in LL-2-CM, BMDM culture medium, and 50 $\mathrm{\%}$ LL-2-CM incubated with BMDMs. *P* values were calculated by one-way ANOVA and Tukey test

The secretion of cytokines was another indicator of macrophage polarization. IL-1 $\upbeta$, IL-6, and TNF-$\mathrm{\alpha }$ were regarded as pro-inflammatory cytokines, while IL-4 and IL-10 were anti-inflammatory cytokines [4, 18–21]. Our data showed that concentrations of IL-6 and TNF-$\mathrm{\alpha }$ were significantly elevated after LL-2-CM stimulation, while IL-1 $\upbeta$, IL-4, and IL-10 were not altered (Fig. 3A-E). It seems paradoxical that markers on cell surface showed anti-inflammatory phenotypes (Fig. 2A, B), while secreted cytokines showed pro-inflammatory phenotypes (Fig. 3A-E). This phenomenon inspired us to dive in the underlying mechanisms of transcriptome changes in BMDMs elicited by LL-2-CM.

### RNA-seq analysis revealed many altered GO terms and KEGG pathways by LL-2-CM

**Fig. 4:**
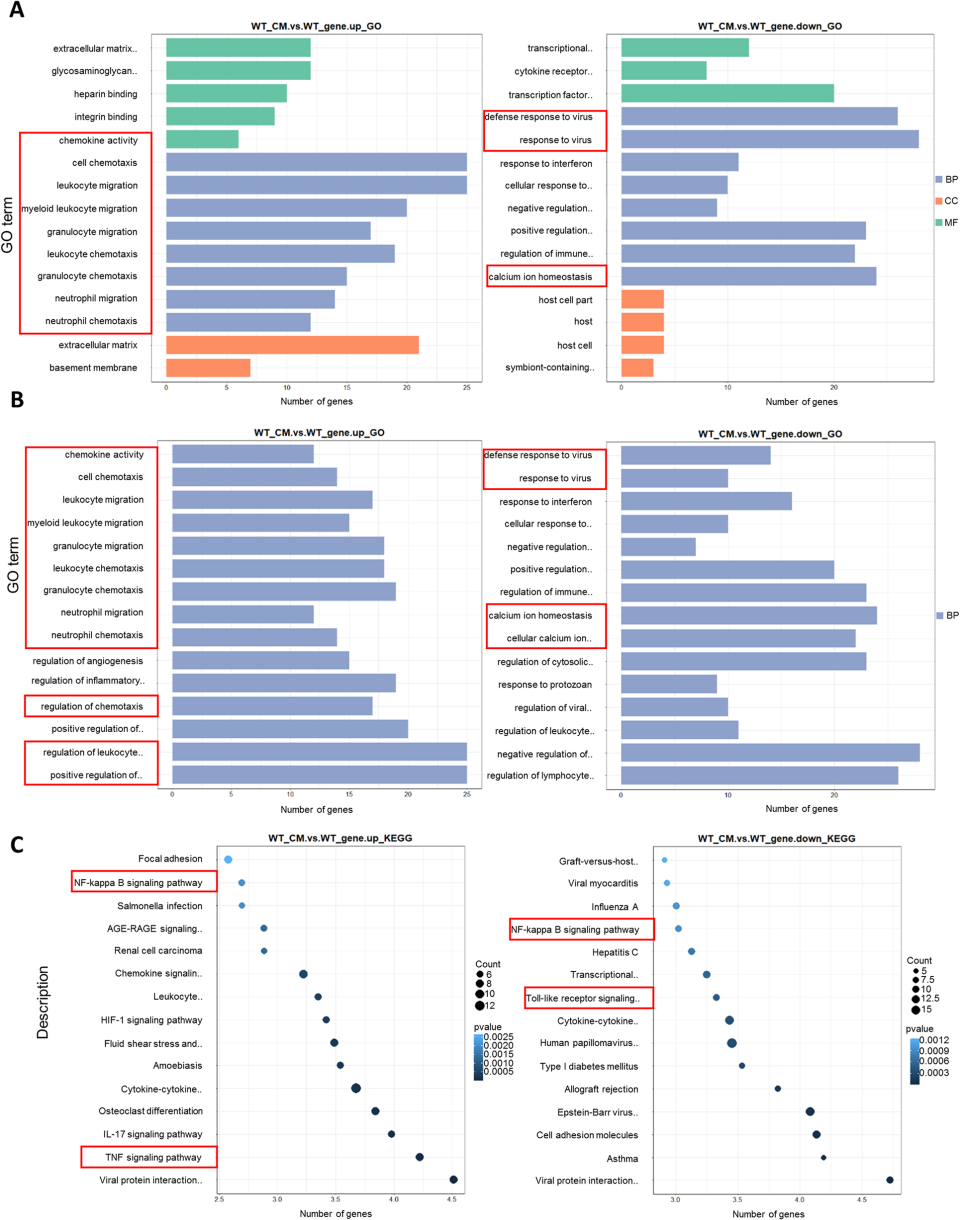
RNA-seq analysis revealed many altered GO terms and KEGG pathways by LL-2-CM. BMDMs cultured with or without LL-2-CM were collected for RNA-seq analysis. The DEGs were annotated by GO and KEGG database. Up regulated and down regulated GO terms including biological process, cellular component, and molecular function **A**. Up regulated and down regulated GO terms including biological process **B**. Up regulated and down regulated KEGG pathways **C**

In order to comprehensively understand the effects of LL-2-CM on BMDMs, RNA-seq was carried out. The data were clustered (Fig. S2). In GO enrichment analysis, many GO terms related with cell chemotaxis and migration were up regulated including cell chemotaxis (GO: 0,060,326), which was the mostly enriched biological process (Fig. 4A, B). This result was in accordance with our scratch and transwell test (Fig. 1D, E). Moreover, many GO terms related with inflammatory response were down regulated including defense response to virus (GO: 0,051,607), which was the mostly enriched biological process (Fig. 4A, B). This result coincided with our flow cytometry data (Fig. 2). In KEGG pathway enrichment analysis, many pathways related with cytokines and migration were up regulated, while pathways related with inflammatory response were down regulated (Fig. 4C). In addition, many pathways related with inflammation and cell adhesion were down regulated.

Interestingly, NF-κB signal pathway (KEGG: mmu04064) was enriched in both up regulated and down regulated list (Fig. 4C). At the same time, many GO terms related with calcium ion homeostasis were down regulated, such as calcium ion homeostasis (GO: 0,055,074), cellular calcium ion homeostasis (GO: 0,006,874), and regulation of cytosolic calcium ion concentration (GO: 0,051,480) (Fig. 4B). Knowing that canonical NF-κB promoted inflammatory response of macrophages [22] and that cytosolic calcium is a stimulator of NF-κB [16], we tried to dissect the influence of LL-2-CM on NF-κB signal in BMDMs and the role of cytosolic calcium in NF-κB signal in further experiment.

Other DEGs, altered GO terms, and altered KEGG pathways have been listed in Supplementary Material.

### LL-2-CM decreased cytosolic calcium in BMDMs

**Fig. 5:**
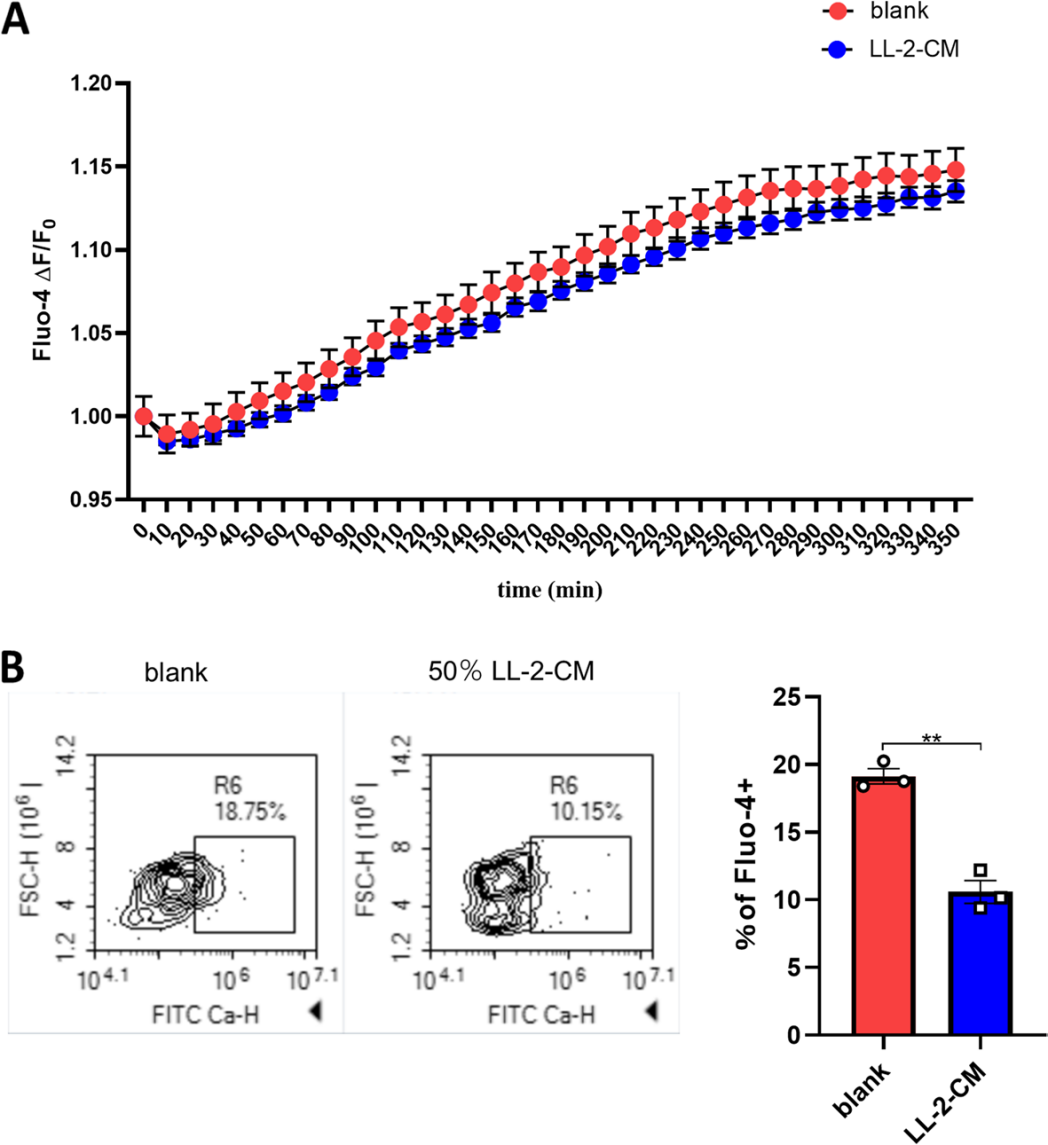
LL-2-CM decreased cytosolic calcium in BMDMs. BMDMs cultured with or without LL-2-CM were stained by cytosolic calcium probe Fluo-4-AM. The short-term alteration of cytosolic calcium within 6 h was detected by fluorescent plate reader **A**. The long-term alteration of cytosolic calcium after 48 h was detected by FCM **B**. *P* values were calculated by Student’s t-test

In RNA-seq, we found three down regulated calcium channels or transporters in GO terms related with cellular calcium ion homeostasis. Calcium channel, voltage-dependent, P/Q type, alpha 1A subunit (*Cacna1a*, GeneID: 12,286) and calcium channel, voltage-dependent, N type, alpha 1B subunit (*Cacna1b*, GeneID: 12,287) are calcium channels through which extracellular calcium enters cytoplasm. However, ATPase, Ca^2+^ transporting, plasma membrane 3 (*Atp2b3*, GeneID: 320,707) is an ATPase that excretes cytosolic calcium against very large concentration gradients. In order to explore whether cytosolic calcium was elevated or reduced by LL-2-CM, we used cytosolic calcium probe, Fluo-4-AM. Within the first 6 h, cytosolic calcium in LL-2-CM treated BMDMs was slightly lower than control group (Fig. 5A). The cytosolic calcium in LL-2-CM treated BMDMs was significantly reduced after 48 h (Fig. 5B). Our data showed that although both entrances and exits of cytosolic calcium in BMDMs were down regulated by LL-2-CM, cytosolic calcium was diminished in general.

### CMT-64-CM affected BMDM polarization and cytosolic calcium in similar pattern

**Fig. 6:**
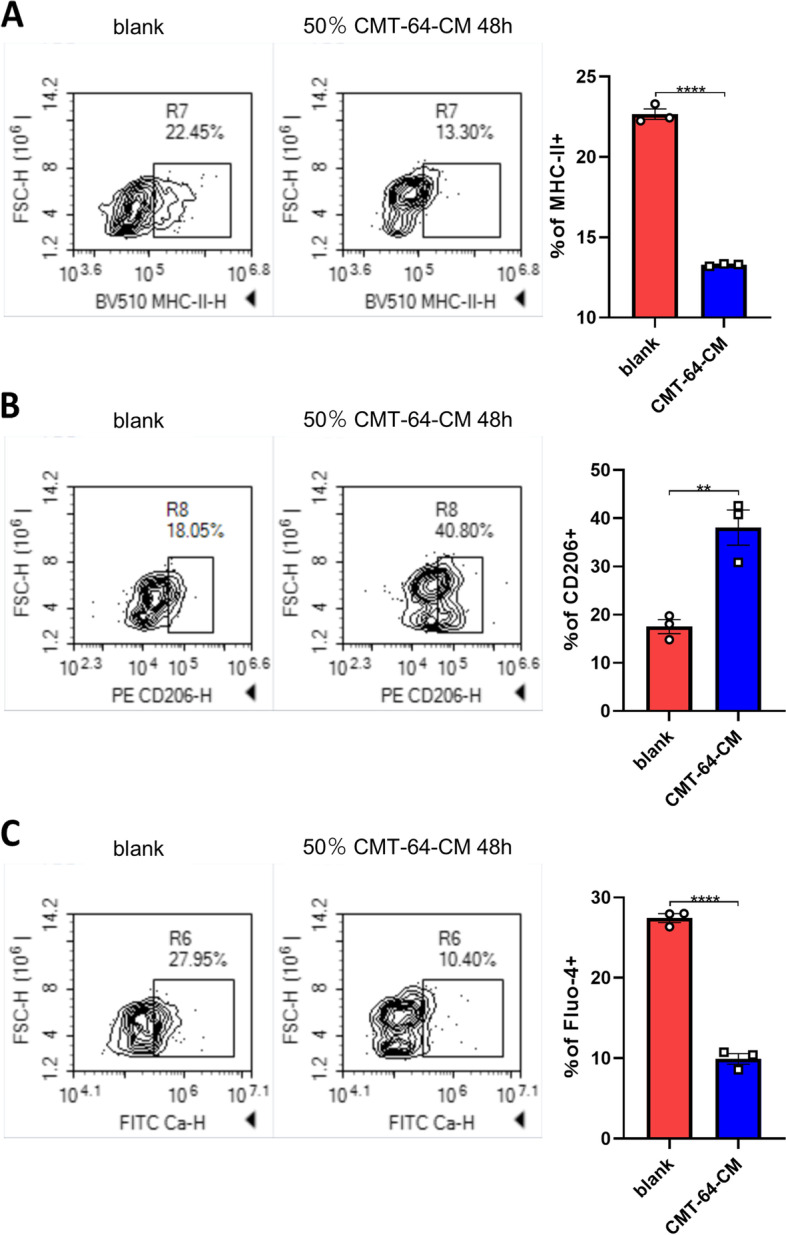
The M2-like polarization and decreased cytosolic calcium was validated in CMT-64-CM treated BMDMs. BMDMs cultured with or without CMT-64-CM were stained by anti-MHC-II **A**, anti-CD206 **B**, and Fluo-4-AM **C**. *P* values were calculated by Student’s t-test

We validated the effects of lung cancer cells conditioned medium on BMDMs by CMT-64. BMDMs cultured in CMT-64-CM showed elevated CD206, attenuated MHC-II, and diminished cytosolic calcium after 48 h (Fig. 6A-C). This data illustrated that the influences of lung cancer cells conditioned medium on BMDMs are repeatable in different cell lines.

### NF-κB signals were regulated by LL-2-CM through cytosolic calcium

**Fig. 7:**
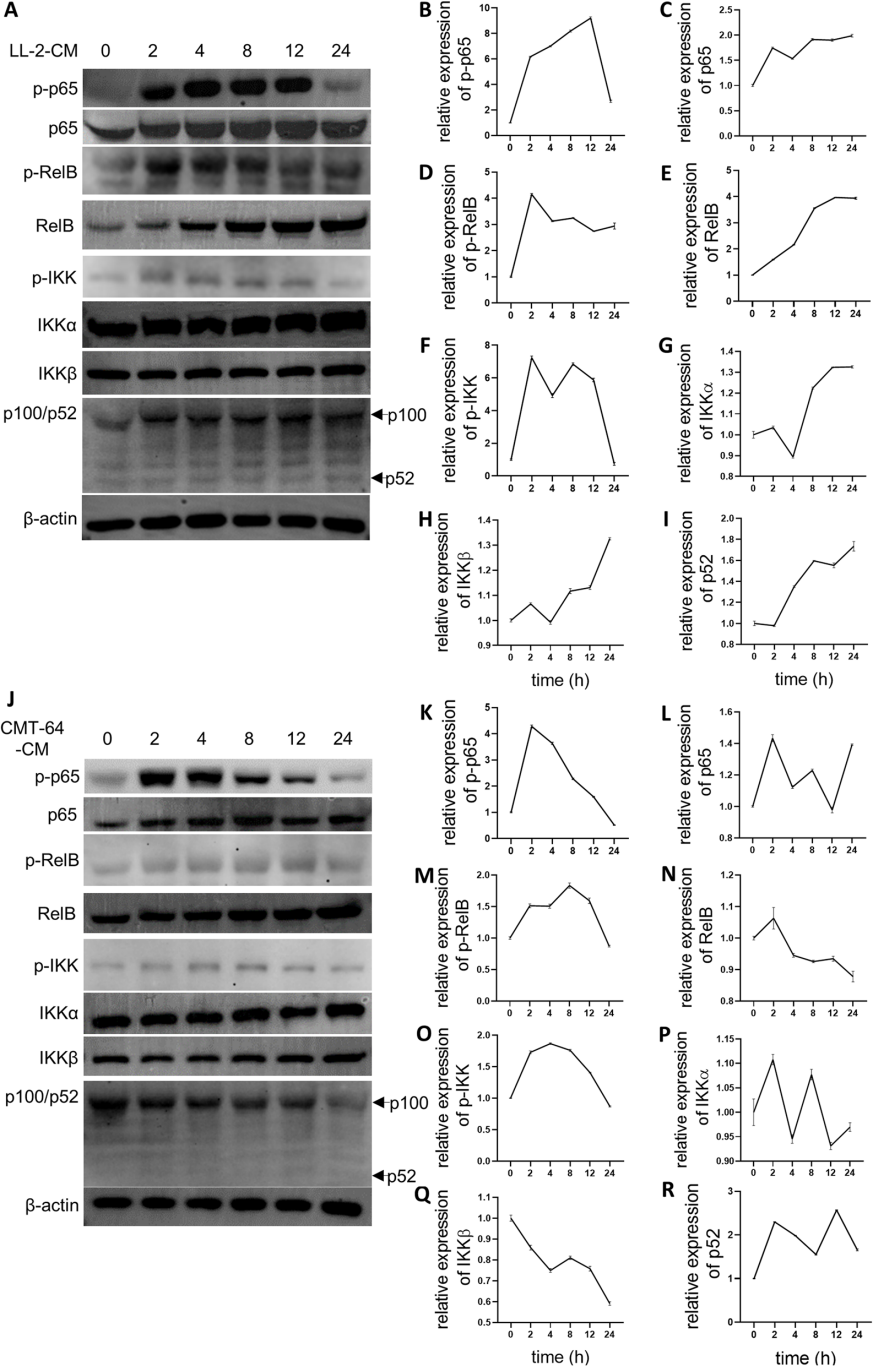
NF-κB signals were regulated by LL-2-CM and CMT-64-CM. BMDMs cultured in LL-2-CM and CMT-64-CM were harvested at different time points for western blot analysis. Dynamic alterations of canonical NF-κB (p-p65 and p65), non-canonical NF-κB (p-RelB, RelB, and p100/p52), and overall NF-κB (p-IKK, IKK $\mathrm{\alpha }$, and IKK $\upbeta$) in LL-2-CM treated BMDMs **A-I** and CMT-64-CM treated BMDMs **J-R** were shown. Full-length blots are presented in Supplementary Fig. 3 and Supplementary Fig. 4

It’s an interesting result that NF-κB signal pathway was enriched in both up regulated and down regulated list. Toll-like receptor signaling pathway (KEGG: mmu04620) was also down regulated accompanying with NF-κB signal pathway, indicating canonical NF-κB signal was inhibited, because TLRs has been shown to elicit canonical NF-κB signal [14, 22]. On the contrary, the up regulated TNF signaling pathway (KEGG: mmu04668) is known to activate both non-canonical and canonical NF-κB signal [23]. Western blot assay was applied to investigate the activation of NF-κB signal pathway. We found that canonical NF-κB signal, indicated by p-p65, was stimulated in the initial 12 h, however it was obviously dampened after 24 h (Fig. 7A, B). In contrast, non-canonical NF-κB signal, indicated by p-RelB, was consistently activated (Fig. 7A, D). Although the expression of p-RelB in 24 h was also slightly lower than that in 2 h, 4 h, and 8 h, it did not decrease dramatically. Additionally, the activation pattern of p-IKK, the up-stream protein of p-p65 and p-RelB, resembled that of p-p65 (Fig. 7A, F). Another molecular involving in non-canonical NF-κB signal, p52, was also consistently elevated by LL-2-CM (Fig. 7A, I).

We also investigated the effect of CMT-64-CM on NF-κB signal pathway in BMDMs. More rapid activation and regression of both canonical (p-p65) and non-canonical (p-RelB and p52) NF-κB signal markers were observed (Fig. 7J, K, M, and R) comparing with that of LL-2-CM. But the phenomenon that promotion of p-p65 was earlier than p-RelB was in accordance with the results from LL-2-CM treated BMDMs.

**Fig. 8:**
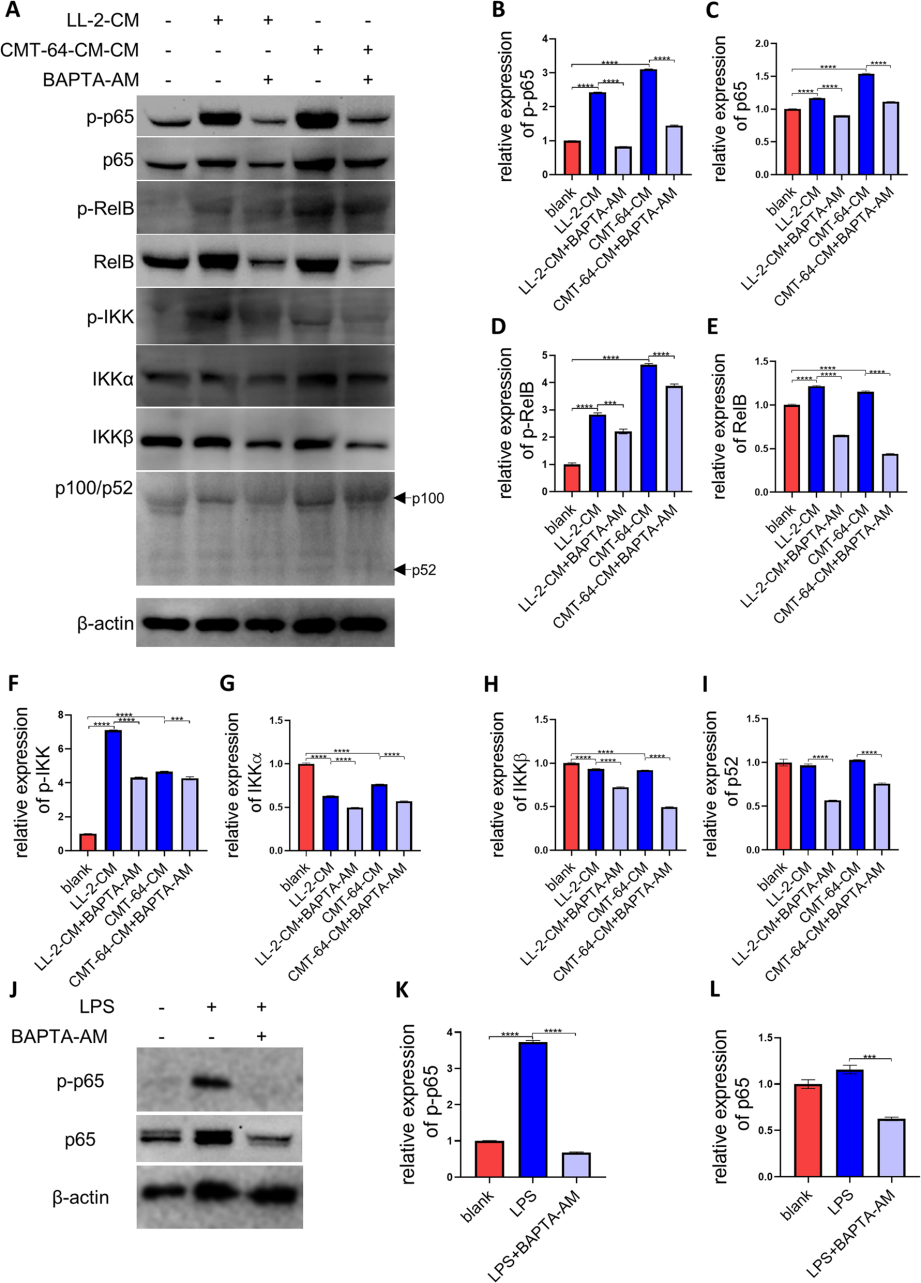
Cytosolic calcium participated in conditioned medium-mediated NF-κB signal and LPS-mediated canonical NF-κB signal. BMDMs treated by LL-2-CM and CMT-64-CM with or without BAPTA-AM for 2 h were collected for western blot. Canonical NF-κB (p-p65 and p65), non-canonical NF-κB (p-RelB, RelB, and p100/p52), and overall NF-κB (p-IKK, IKK $\mathrm{\alpha }$, and IKK $\upbeta$) were shown and calculated **A-I**. LPS elicited canonical NF-κB in BMDMs, which was suppressed by BAPTA-AM **J-L**. Full-length blots are presented in Supplementary Fig. 5 and Supplementary Fig. 6. *P* values were calculated by one-way ANOVA and Tukey test

In order to dissect the relationship between down-regulated NF-κB signal and diminution of cytosolic calcium, we tested whether chelating cytosolic calcium could suppress LL-2-CM and CMT-64-CM-elicited NF-κB signal. Our result showed that BAPTA-AM, a specific and quick cytosolic calcium chelator [24, 25], suppressed both canonical and non-canonical NF-κB signal elicited by LL-2-CM and CMT-64-CM early at 2 h (Fig. 8A-I). Interestingly, canonical NF-κB indicated by p-p65 seemed to be more susceptible to BAPTA-AM treatment than non-canonical NF-κB indicated by p-RelB (Fig. 8B, D). These results demonstrated that deprivation of LL-2-CM and CMT-64-CM-induced NF-κB signal could be achieved early through acute sequestration of cytosolic calcium, which built the positive link between NF-κB signal and cytosolic calcium.

### LPS-mediated canonical NF-κB signal was depended on cytosolic calcium

Both cytosolic calcium and canonical NF-κB were decreased after 24 h, which inspired us to further evaluate the relationship between cytosolic calcium and canonical NF-κB signal. LPS, a ligand of TLR4, is known to elicit canonical NF-κB signal and M1-like polarization of macrophages [14, 22]. We applied LPS to specifically provoke canonical NF-κB signal in BMDMs. Our result revealed that LPS treatment remarkably evoked expression of p-p65 in BMDMs, while chelating cytosolic calcium with BAPTA-AM significantly abrogated canonical NF-κB signal (Fig. 8J-L). This finding further verified that cytosolic calcium was indispensable for canonical NF-κB signal in macrophages.

### Cytosolic calcium was involved in M1-like polarization of BMDMs evoked by LPS

**Fig. 9:**
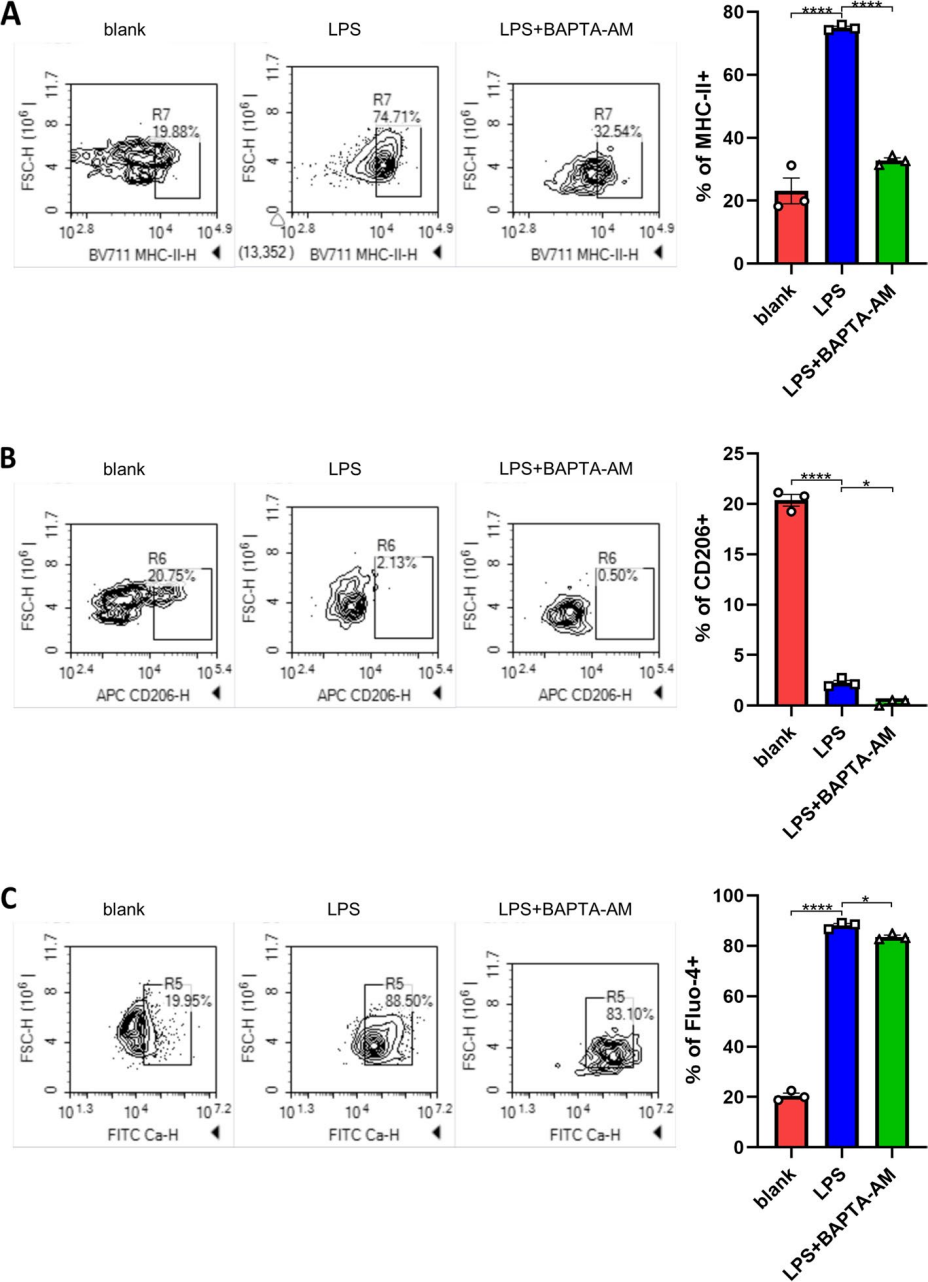
Cytosolic calcium was involved in M1-like polarization of BMDMs evoked by LPS. Untreated BMDMs and BMDMs treated with LPS or LPS plus BAPTA-AM were stained by Fluo-4-AM, anti-MHC-II, and anti-CD206 and analyzed by FCM. Alteration of MHC-II expression **A**. Alteration of CD206 expression **B**. Alteration of cytosolic calcium **(C)**. *P* values were calculated by one-way ANOVA and Tukey test

Since chelating cytosolic calcium significantly inhibited canonical NF-κB signal elicited by LPS, we attempted to explore whether LPS could elevate cytosolic calcium in BMDMs and whether BAPTA-AM could inhibit M1-like polarization of BMDMs evoked by LPS. Flow cytometry data showed that MHC-II was significantly up regulated (Fig. 9A) and CD206 was significantly down regulated (Fig. 9B) by LPS treatment indicating M1-like polarization of macrophages. At the same time, LPS elevated cytosolic calcium (Fig. 9C), indicating that cytosolic calcium participated in M1-like polarization of BMDMs. BAPTA-AM obviously attenuated expression of MHC-II in LPS-treated BMDMs (Fig. 9A), which further substantiated the role of cytosolic calcium in M1-like polarization of LPS-treated BMDMs. Interestingly, BAPTA-AM also decreased expression of CD206 in LPS-treated BMDMs (Fig. 9B). A possible explanation for the decreased CD206 expression is that BAPTA-AM also inhibited non-canonical NF-κB signal (Fig. 8D). The slightly decreased Fluo-4 fluorescence in BAPTA-AM (Fig. 9C) treatment group could be explained by competitive chelation of calcium by both BAPTA-AM and Fluo-4. Together, our results illustrated that cytosolic calcium was the key factor for M1-like polarization of BMDMs.

## Discussion

Many researches have proven that existence and reprogramming of stromal cells, especially macrophages, in TME are crucial for tumor progression [1, 12, 13]. Better understanding of what signal pathways regulate the reprogramming of macrophages and how these signal pathways are evoked would be beneficial for developing novel therapeutic methods against tumor progression.

**Fig. 10:**
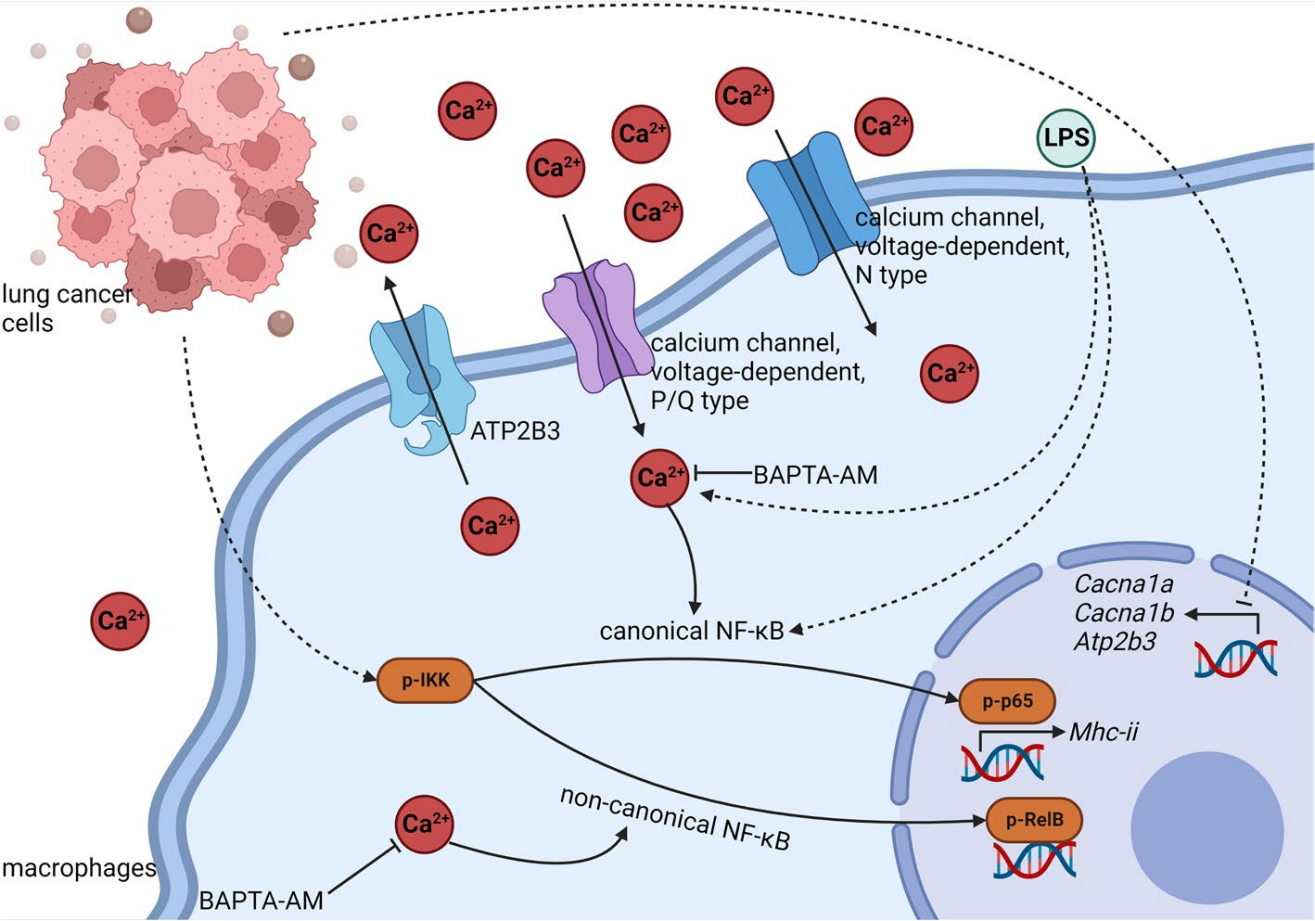
Summarization of the related mechanisms. LL-2-CM inhibited expression of calcium channels and decreased cytosolic calcium concentration, which inhibited activation of NF-κB. On the contrary, LPS was able to elevate cytosolic calcium concentration, elicit canonical NF-κB, and stimulate M1-like polarization of BMDMs. Created with Biorender.com

In this work, we found that LL-2-CM promoted proliferation, migration, and M2-like polarization of macrophages. Treatment of LL-2-CM increased viable macrophages detected by CCK8, implying the potential existence of cytokines responsible for macrophage proliferation in LL-2-CM. Scratch and transwell test found that BMDMs incubated in LL-2-CM showed augmented migration ability. These results substantiated that LL-2 cells are probably able to secrete some factors to boost recruitment and local proliferation of macrophages in TME. The macrophages in TME were further polarized to M2-like macrophages by LL-2-CM stimulation, which would facilitate immune evasion of tumor cells. RNA-seq revealed that LL-2-CM treatment promoted many pathways involved in chemotaxis and migration and inhibited many pathways involved in inflammation, which explained the phenotypes we observed above. We then focused on NF-κB signal, which was up regulated and down regulated simultaneously in KEGG enrichment analysis. Western blot showed that canonical NF-κB signal remarkably decreased at 24 h after LL-2-CM administration, while non-canonical NF-κB signal was constantly activated by LL-2-CM. Interestingly, canonical NF-κB signal and cytosolic calcium were simultaneously reduced by LL-2-CM treatment. Similar dynamic changes of NF-κB signal and macrophage polarization were also observed in CMT-64-CM treated BMDMs. Considering that TLR4 activated canonical NF-κB signal and promoted expression of M1-like markers in macrophages [22] and that calcium was able to activate canonical NF-κB signal in lymphocyte [16], we tried to figure out whether the cytosolic calcium reduction was the cause of dampened canonical NF-κB signal in BMDMs. As expected, LPS, the specific TLR4 stimulator, elevated cytosolic calcium, initiated canonical NF-κB signal, and induced M1-like polarization in BMDMs, which was substantially suppressed by chelating cytosolic calcium via BAPTA-AM. Additionally, sequestration of cytosolic calcium through BAPTA-AM also caused early deprivation of canonical and non-canonical NF-κB signal induced by LL-2-CM and CMT-64-CM. In a word, we found a positive relationship between cytosolic calcium, canonical NF-κB signal, and M1-like phenotype of macrophages. The related mechanisms have been summarized in Fig. 10.

RNA-seq found that genes of three calcium channel/transporter, *Cacna1a, Cacna1b,* and *Atp2b3*, were decreased in LL-2-CM treated BMDMs. Proteins coded by *Cacna1a* and *Cacna1b* are channels for calcium entering cytoplasm, while protein coded by *Atp2b3* is the transporter for excreting cytosolic calcium. With the help of calcium probe, we found that LL-2-CM decreased cytosolic calcium in BMDMs. We speculated that *Cacna1a* and *Cacna1b* might be initially down-regulated genes, and that down-regulation of *Atp2b3* might be the secondary effect caused by decreased cytosolic calcium. In the future, dissecting the crosstalk of different calcium channel/transporter on both protein level and transcriptome level would be a compelling work.

Our data indicated that reduced cytosolic calcium was probably the cause of suppressed canonical and non-canonical NF-κB signal. However it remains fuzzy why the non-canonical NF-κB signal was diminished slower than canonical NF-κB signal in LL-2-CM and CMT-64-CM treated BMDMs. It seems that p-p65 was more susceptible to BAPTA-AM than p-RelB, but the underlying mechanisms need further investigation. The canonical NF-κB signal has been proven to support M1-like polarization of macrophages [6, 22], while the role of non-canonical NF-κB in tumor associated macrophages is not well illustrated. It seems that non-canonical NF-κB inhibited inflammatory response in dendritic cells [26] and facilitated drug resistance in TAMs [27]. On the contrary, another research demonstrated that non-canonical NF-κB participated in M1-like polarization of macrophages induced by baicalin [28]. Whether non-canonical NF-κB was involved in other mechanisms for immune evasion is still unknown. Future researches answering these questions may help with establishing a better understanding of the crosstalk between tumor cells and macrophages. Another interesting finding is the different dynamic changes of canonical and non-canonical NF-κB signal. Previous results declare that macrophages may express M1-like phenotypes at early stage and they would be repolarized toward M2-like phenotypes [11]. This is in accordance with our findings including production of pro-inflammatory IL-6 and TNF-$\mathrm{\alpha }$, slightly elevated MHC-II in 24 h, and slightly decreased CD206 in 24 h by LL-2-CM treatment. Although the pro-inflammatory phenotypes may facilitate anti-tumor immunity [18, 22], immuno-suppressive myeloid-derived suppressor cells are also recruited in TME by these cytokines, which caused suppressed anti-tumor immunity and drug resistance [29–31]. Thus the cross-talk between cancer cells and macrophages is much more complicated beyond merely suppression anti-tumor immunity.

Our work provided an overview of the effects of LL-2-CM on BMDMs through both bioinformatics and experiment. The RNA-seq data revealed many altered pathways after LL-2-CM treatment, among which some pathways might be the potential targets for therapy. For example, elevating cytosolic calcium through inhibiting ATP2B3 or activating the voltage-dependent calcium channels in macrophages might start M1-like polarization and enhance anti-tumor immunity. Whether it is feasible to manipulate NF-κB signal and phenotypes of TAMs through activators and inhibitors of calcium channels is a tempting question.

## Conclusion

In conclusion, our study elucidated that LL-2-CM facilitated proliferation, migration, and M2-like polarization of BMDMs. During this process, reduced cytosolic calcium was the possible cause of the suppression of pro-inflammatory canonical NF-κB signal. This mechanism was further validated in CMT-64-CM treated BMDMs. Development of future interventions could focus on elevating cytosolic calcium in macrophages.

## Supplementary Information

**Additional file 1:** **Supplementary Fig. 1.** The gating strategies for flow cytometry. Gating strategy for figure 2**A**. Gating strategy for figure 5**B**. Gating strategy for figure 6 and figure 9**C**.

**Additional file 2:** **Supplementary Fig. 2.** The DEGs of the six group were clustered.

**Additional file 3:** **Supplementary Fig. 3.**Full-length blots of Figure 7A.

**Additional file 4:** **Supplementary Fig. 4.** Full-length blots of Figure 7J.

**Additional file 5:** **Supplementary Fig. 5.** Full-length blots of Figure 8A.

**Additional file 6:** **Supplementary Fig. 6.** Full-length blots of Figure 8J.

**Additional file 7:** **Supplementary Table 1.** up-regulated genes.

**Additional file 8:** **Supplementary Table 2.** down-regulated genes.

**Additional file 9:** **Supplementary Table 3.** up-regulated GO.

**Additional file 10:** **Supplementary Table 4.** down-regulated GO.

**Additional file 11:** **Supplementary Table 5.** up-regulated KEGG.

**Additional file 12:** **Supplementary Table 6.** down-regulated KEGG.

## Data Availability

The related data and material in the section of my manuscript is available. When reasonably requested, the data set used and/or analyzed in the current study can be obtained from the authors. The datasets generated and/or analysed during the current study are available in the GEO repository (record GSE210767). The following secure token has been created to allow review of record GSE210767 while it remains in private status: ijodoqyovhczpot. Please contact Ziqi Zhang via zhangziqicd@126.com if our data is required for appropriate reason.

## Abbreviations

TME: Tumor microenvironment
NF-κB: Nuclear factor κB
LL-2-CM: LL-2-derived conditional medium
BMDMs: Bone marrow-derived macrophages
CMT-64-CM: CMT-64-derived conditioned medium
IL-6: Interleukin-6
TNF-$\mathrm{\alpha }$: Tumor necrosis factor-α
LPS: Lipopolysaccharide
TLRs: Toll-like receptors
IFN-γ: Interferon gamma
MHC-II: Major histocompatibility complex class II
iNOS: Inducible nitric oxide
IL-4: Interleukin-4
IL-10: Interleukin-10
TGF-β: Transforming growth factor beta
NKs: Nature killer cells
CSFR: Colony stimulating factor receptor
ATCC: American Tissue Type Culture Collection
DEGs: Differentially expressed genes
FC: Fold change
GO: Gene Ontology
KEGG: Kyoto Encyclopedia of Genes and Genomes
Cacna1a: Calcium channel, voltage-dependent, P/Q type, alpha 1A subunit
Cacna1b: Calcium channel, voltage-dependent, N type, alpha 1B subunit
ATP2b3: ATPase, Ca2 + transporting, plasma membrane 3
