# Supplementary figures and images for: Tumor cells-derived conditioned medium induced pro-tumoral phenotypes in macrophages through calcium-nuclear factor κB interaction
Source: BMC Cancer. 2022 Dec 19;22:1327. doi: 10.1186/s12885-022-10431-8 (PMC9762082; doi:10.1186/s12885-022-10431-8)

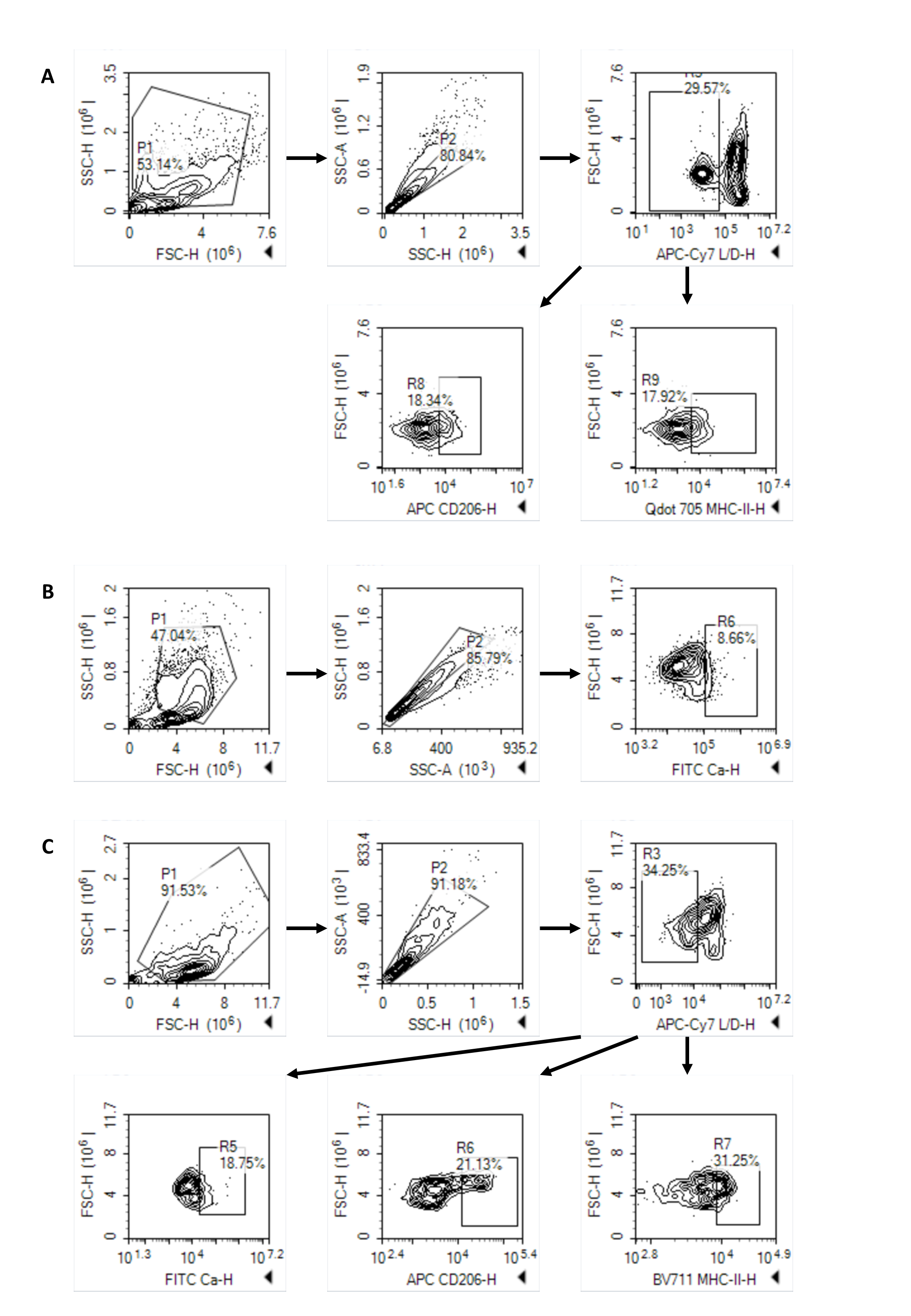

Supplement: Supplementary file 4 — Additional file 4: Supplementary Fig. 4. Full-length blots of Figure 7J. [file 12885_2022_10431_MOESM4_ESM.tif]

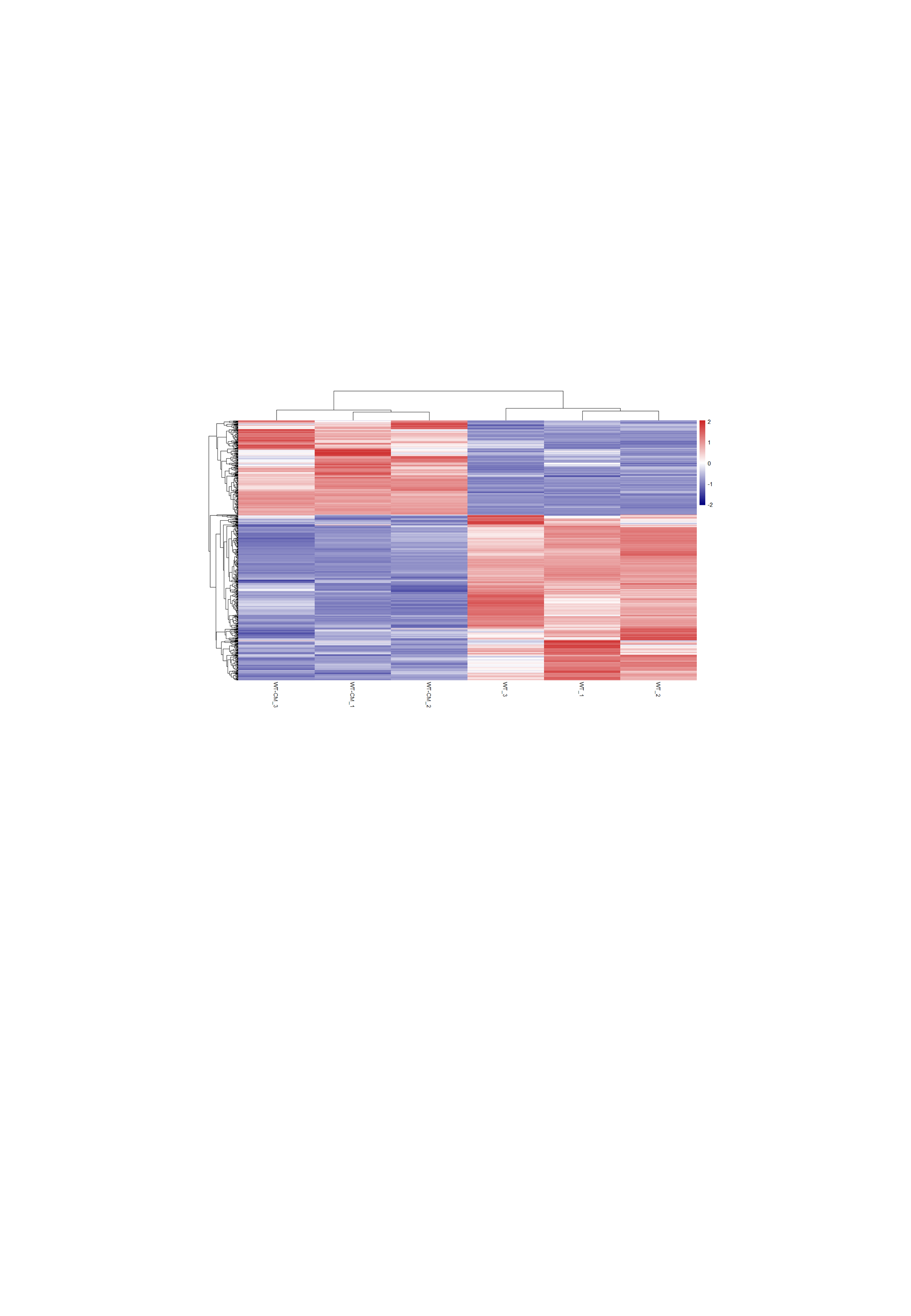

Supplement: Supplementary file 5 — Additional file 5: Supplementary Fig. 5. Full-length blots of Figure 8A. [file 12885_2022_10431_MOESM5_ESM.tif]

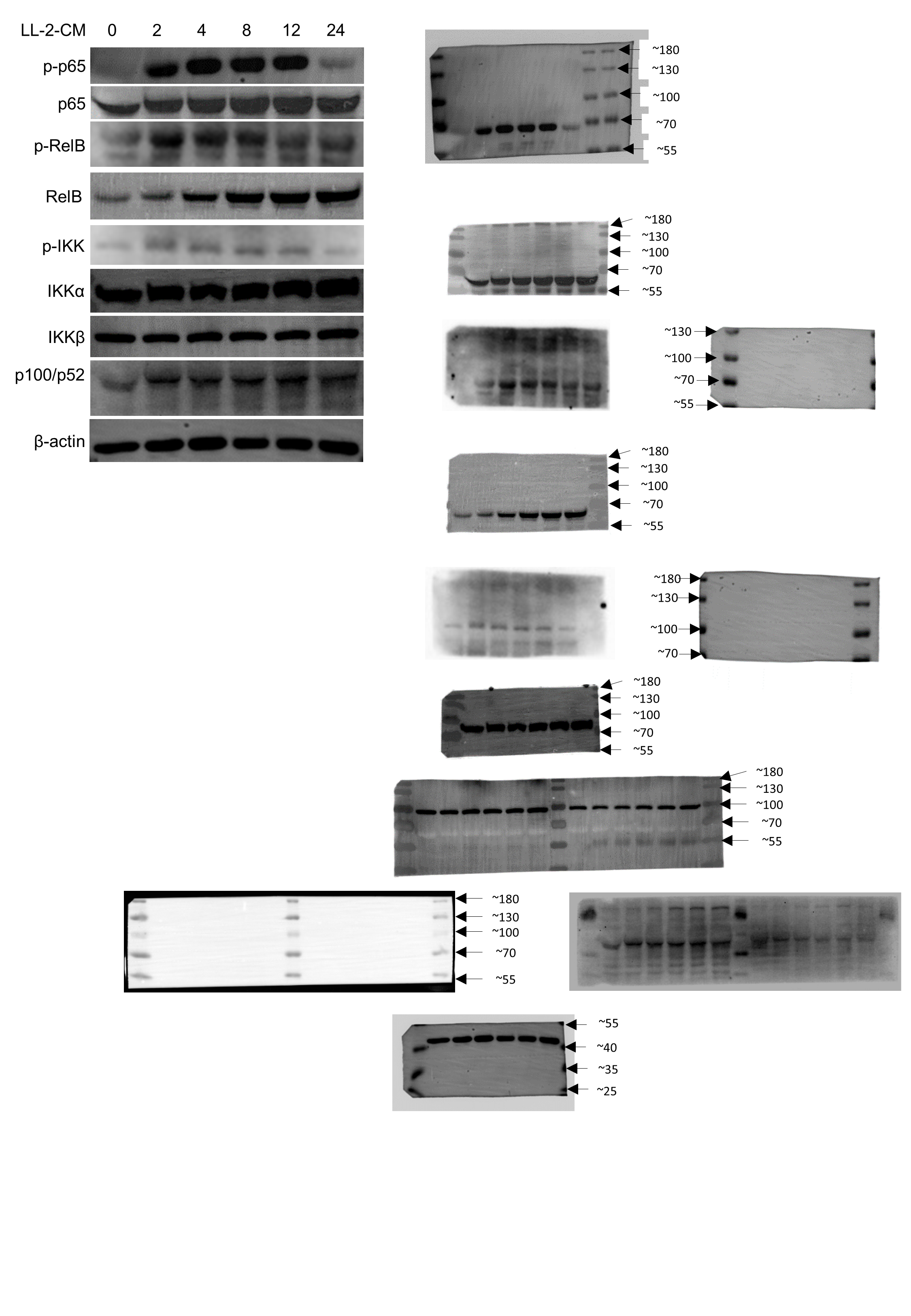

Supplement: Supplementary file 6 — Additional file 6: Supplementary Fig. 6. Full-length blots of Figure 8J. [file 12885_2022_10431_MOESM6_ESM.tif]

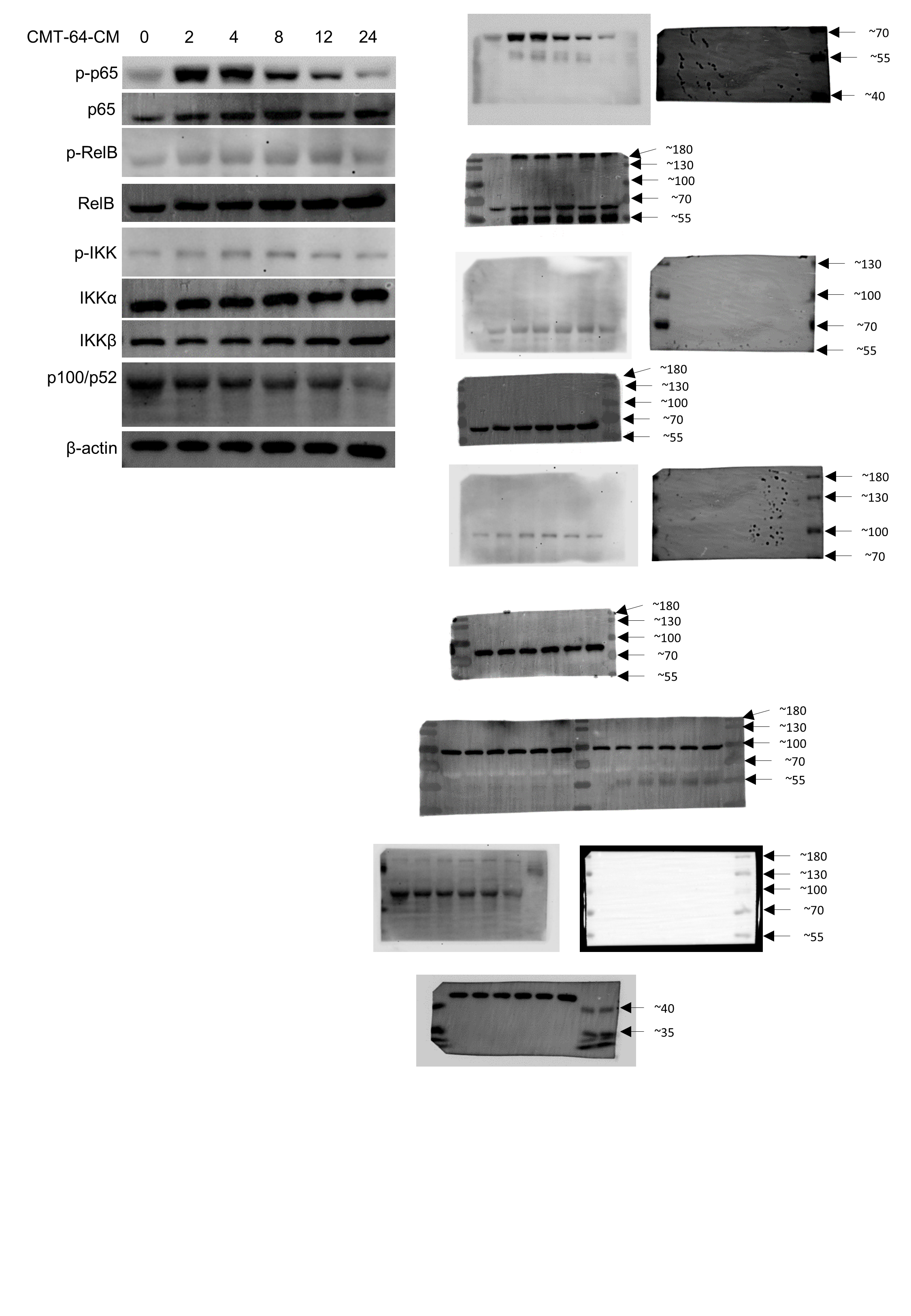

Supplement: Supplementary file 7 — Additional file 7: Supplementary Table 1. up-regulated genes. [file 12885_2022_10431_MOESM7_ESM.tif]

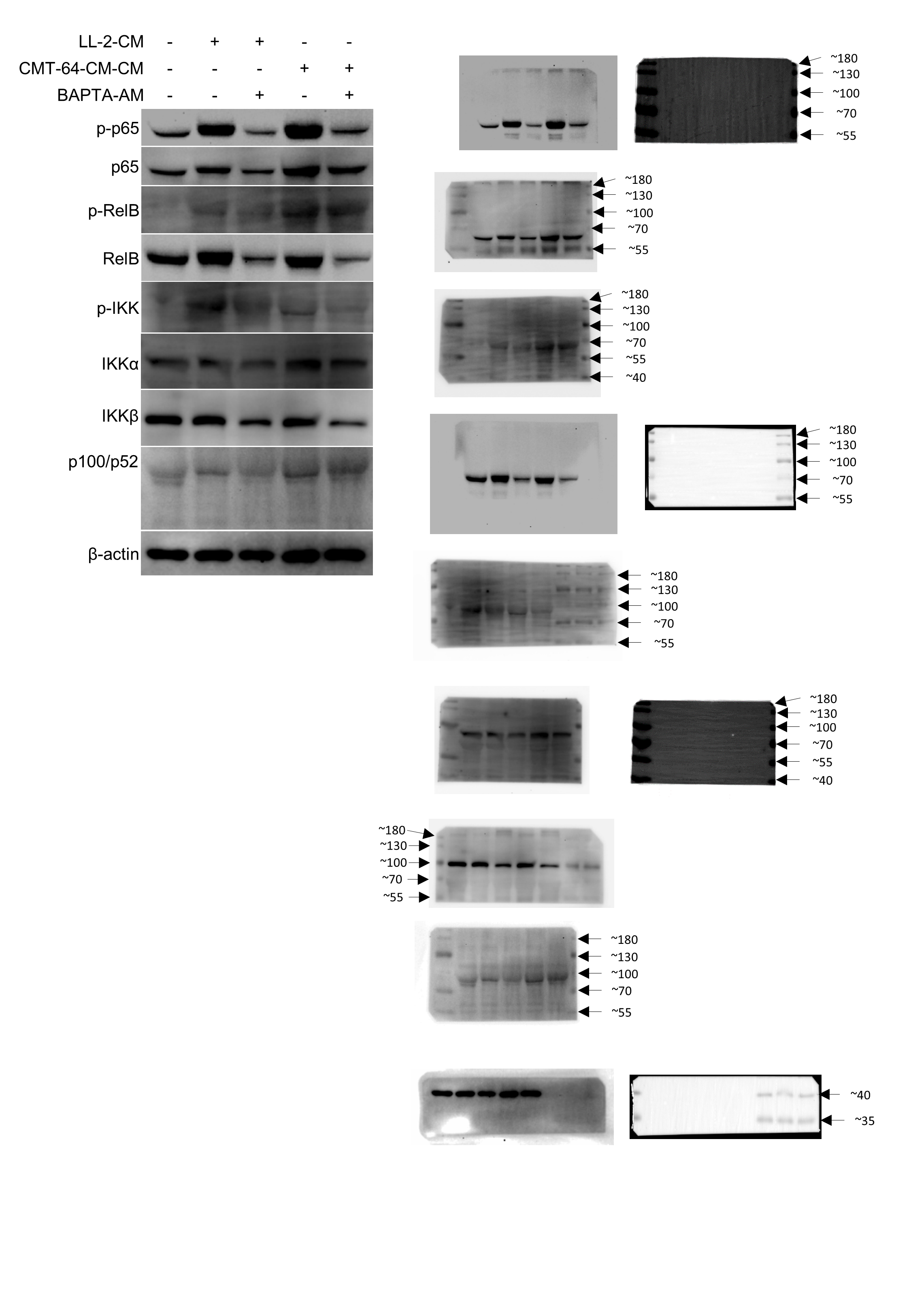

Supplement: Supplementary file 8 — Additional file 8: Supplementary Table 2. down-regulated genes. [file 12885_2022_10431_MOESM8_ESM.tif]

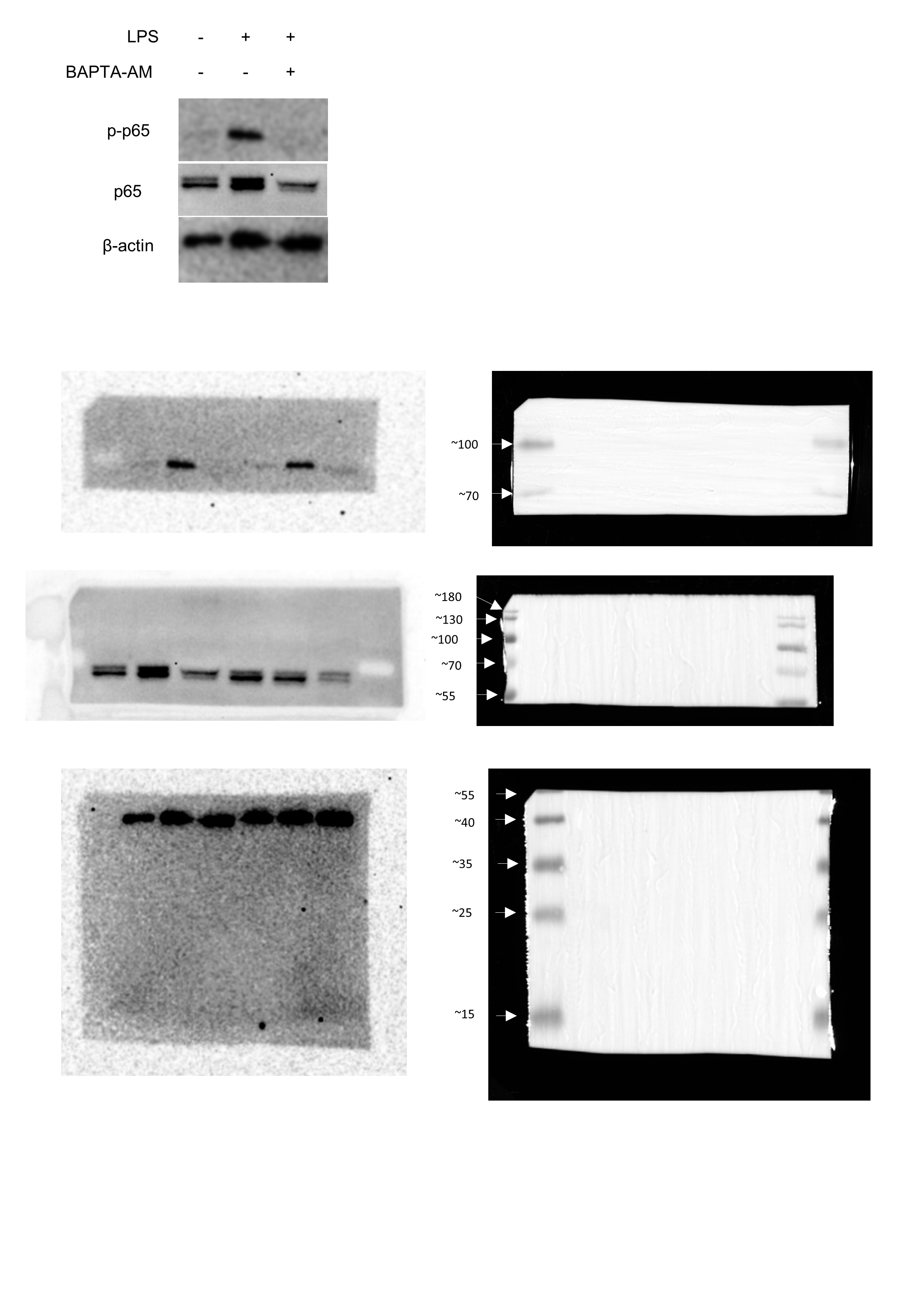

Supplement: Supplementary file 9 — Additional file 9: Supplementary Table 3. up-regulated GO. [file 12885_2022_10431_MOESM9_ESM.tif]
